# Artificial intelligence in surgical care within low-income and middle-income countries: a scoping review of development, validation, and deployment

**DOI:** 10.1016/j.eclinm.2026.103836

**Published:** 2026-03-16

**Authors:** Aashobanaa Duraisaminathan Valli, Samuel James Tingle, Sofia Kazerouni, Tanissha Sanjay Raj Kalpana, Bishow Karki, Stephen R. Knight, Colin Wilson, Georgios Kourounis

**Affiliations:** a Translational and Clinical Research Institute, Newcastle University, Newcastle upon Tyne, UK; b NIHR Blood and Transplant Research Unit at Cambridge and Newcastle Universities, Newcastle upon Tyne, UK; c Institute of Transplantation, The Freeman Hospital, Newcastle upon Tyne, UK; d Devarajan Medical Centre, Chennai, Tamil Nadu, India; e Leeds Teaching Hospitals NHS Trust, Leeds, UK; f Pulchowk Campus, Tribhuvan University, Lalitpur, Nepal; g Queen Elizabeth University Hospital, Glasgow, UK; h Health Economics and Health Technology Assessment, School of Health and Wellbeing, Clarice Pears Building (Level 3), University of Glasgow, Glasgow, UK

**Keywords:** Artificial intelligence, Surgical procedures, Developing countries, Global health, Health information systems, Medical informatics

## Abstract

Artificial intelligence (AI) has the potential to expand access to high-quality surgical care in low-income and middle-income countries (LMICs), yet the extent and maturity of AI research in these settings remain unclear. We conducted a prospectively registered scoping review (osf.io/9PV6A) to synthesize primary evidence on the use of AI in LMIC surgical care. PubMed, Scopus, and Web of Science were searched for studies evaluating AI in surgical contexts within LMICs up to July 14, 2025. From 2602 records, 475 studies met inclusion criteria. Most were conducted in upper-middle-income countries (n = 376, 79·1%), with the overwhelming majority from China (n = 305, 64·2%). Only 46 studies (9·7%) were conducted in lower-middle-income countries and 5 (1·1%) in low-income countries. Research was predominantly retrospective (68%), and only nine randomised controlled trials were identified (2%). Most studies focused on model development (67%), with few reporting external validation (30%) or clinical deployment (3%), mostly as pilot trial-based integrations. Barriers to AI implementation included fragmented data systems, limited infrastructure, and workforce constraints. Facilitators included widespread smartphone access and growing international collaborations. Despite rapid growth, AI research remains in the early stages of development. Focus on model accuracy alone is insufficient if health systems lack the capacity for adoption and integration.

## Introduction

Artificial intelligence (AI) refers to computer systems designed to learn patterns from data and perform specific tasks without being explicitly programmed with fixed rules. In contemporary clinical research, this typically includes machine learning and deep learning approaches that are trained on labelled or unlabelled datasets and whose performance is evaluated empirically on new data.^1^, ^2^, ^3^, ^4^ In medicine, AI has become a key driver of innovation, aiming to enhance diagnostics, improve image analysis, support clinical decision making, and optimise patient care.^5^, ^6^, ^7^ Its diverse applications extend across multiple specialties including radiology, pathology, surgery, and broader areas such as public health.^5^^,^^6^^,^^8^

One of AI's potential benefits is its capacity for remote deployment, offering the opportunity to expand access to high-quality care in resource-limited health systems.^9^ This capability holds particular relevance for low-income and middle-income countries (LMICs), where financial constraints, limited infrastructure, workforce shortages, and geographic barriers often hinder access to medical and surgical care.^10^^,^^11^ In these settings, AI could contribute to improvements in service delivery and patient outcomes.

Although AI research has grown rapidly, particularly in high-income countries (HICs), its development, validation, and deployment in surgical care within LMICs remain comparatively limited.^11^^,^^12^ As AI's potential to improve surgical care in LMICs gains more attention, understanding its real-world potential benefits is essential.^13^ While several reviews and editorials have addressed AI in global health or surgical contexts, there has been no systematic synthesis of primary studies examining AI development, validation, and deployment within surgical care across LMIC settings.^12^ Addressing this gap is important for capturing existing innovations, evaluating outcomes, and informing future practice in global surgery research.

Given the heterogeneous and predominantly early-stage nature of research on AI in LMIC surgical care, a scoping review provides the appropriate method to map and synthesise the available evidence. The primary aim of this review is to characterise the scope, design, and maturity of published studies. Secondary aims are to describe geographical and specialty distributions, summarise reported outcomes, and identify barriers and facilitators shaping the progress towards clinical deployment.

## Methods

### Search strategy and selection criteria

We conducted a scoping review following Preferred Reporting Items for Systematic Reviews and Meta-Analyses extension for Scoping Reviews (PRISMA-ScR) guidelines.^14^ This methodology was selected to enable a broad synthesis of the existing literature. The protocol was registered prospectively with the Open Science Framework (osf.io/9pv6a) on 18/03/2025.^15^

We searched PubMed, Scopus, and Web of Science for studies reporting on the implementation of AI in surgical care within LMICs. Implementation was used as an umbrella term to include model conceptualisation and development, internal validation, external validation, and clinical deployment. Clinical deployment was defined as use within a real patient care pathway in which AI outputs were available at the point of care and capable of informing a clinical action. Searches were performed from database inception to July 14, 2025. The strategy combined terms relating to surgery, artificial intelligence, and LMIC settings, with the full search strategies for each database detailed in Appendix 1 of the supplementary file. In addition, citation and reference lists of identified review articles were hand searched to capture further relevant studies not retrieved by the main search.

Countries were classified as low-income and middle-income using the 2025 World Bank threshold of US$14,005 (£11,120) gross national income per capita.^16^ We additionally verified that all included studies corresponded to periods in which the country of study met LMIC classification at the time of publication. We included studies involving surgical patients or surgical care in LMIC settings, as well as studies with mixed LMIC and HIC populations. Eligible interventions comprised the use of AI technologies in any aspect of surgical care, including preoperative planning, intraoperative support, postoperative care, surgical education, and multimodal or perioperative AI models that integrated radiological or histological data alongside clinical inputs. All study designs were considered, and no comparator was required. Only papers published in English were reviewed.

We excluded studies focused exclusively on HIC populations, as well as those describing robotic surgery without an AI component, simulation or virtual reality tools without AI integration, or prediction models limited to classical statistical methods. Studies using exclusively radiological or histological image data as inputs were also excluded. Protocols, meeting abstracts, commentaries, editorials, opinion pieces without primary data, bibliometric analyses, review articles, retracted articles, and preprints were excluded (see Supplementary Table S1 for full eligibility criteria).

### Data extraction

Following deduplication, two reviewers (ADV and GK) independently screened titles and abstracts against the predefined eligibility criteria, with disagreements resolved by discussion or referral to a third reviewer (SJT). To accommodate timely analysis of the large number of included studies, all eligible study abstracts were exported into a comma-separated values (CSV) file and were processed using a large language model (Claude Sonnet 4·0, via GitHub Copilot), which applied a structured extraction protocol. Additional methodological details are provided in Supplementary Material, Appendix 2. Extracted variables included study characteristics, surgical specialty, AI category, surgical care stage, study design, and development stage mapped to the Organisation for Economic Co-operation and Development (OECD) Framework for the Classification of AI Systems.^17^ Each record was then verified against the full text by a human reviewer (ADV), with discrepancies resolved by a second reviewer (GK).

### Qualitative synthesis of barriers and facilitators

In addition to structured data extraction, we conducted a qualitative narrative synthesis of the full texts of studies from lower-income and lower-middle-income countries to identify barriers and facilitators influencing the development and deployment of surgical AI. This restriction was applied a priori to prioritise settings with the greatest resource constraints, where implementation challenges are often distinct and may be obscured if synthesised across heterogeneous LMIC contexts. Findings were grouped into broad thematic categories (model and data challenges, infrastructure and technology, workforce, and governance). This synthesis was intended to provide contextual insight to the review and inform the discussion, rather than to quantify the frequency of individual themes.

### Role of the funding source

Funding bodies provided salary support only and had no role in the study design, data collection, analysis, interpretation, manuscript preparation, or the decision to submit for publication.

## Results

### Study characteristics

**Fig. 1:**
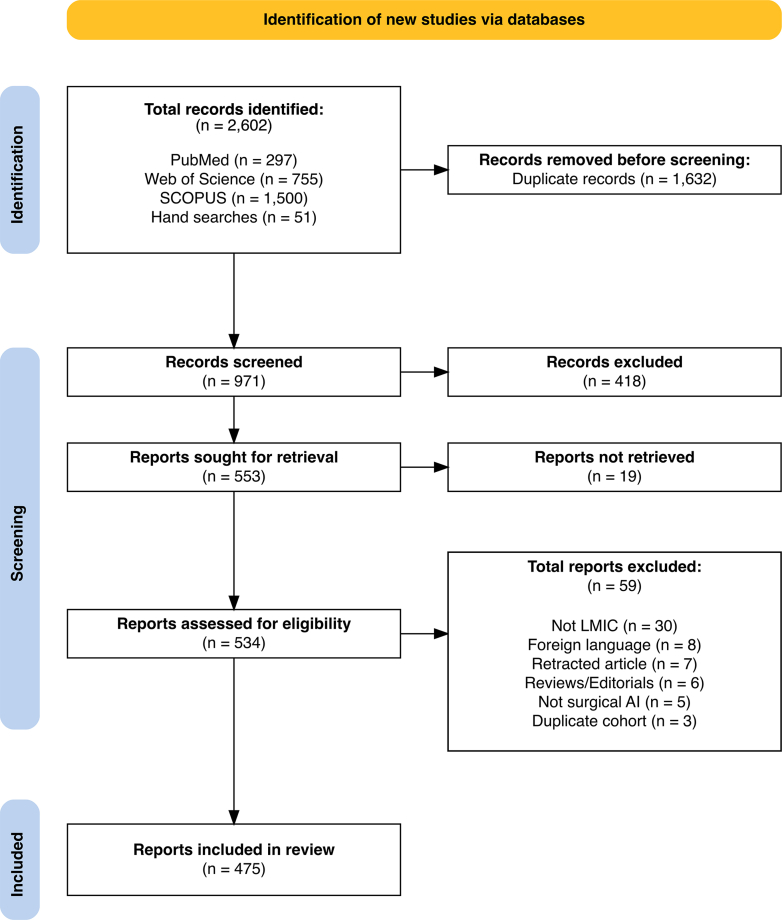
**PRISMA flow diagram outlining the screening and study selection process**.

The search resulted in 971 unique records of which 475 were included in the review. The detailed selection process is outlined in the PRISMA flow diagram (Fig. 1). A complete list of the included articles is provided in the supplementary CSV file.

**Fig. 2:**
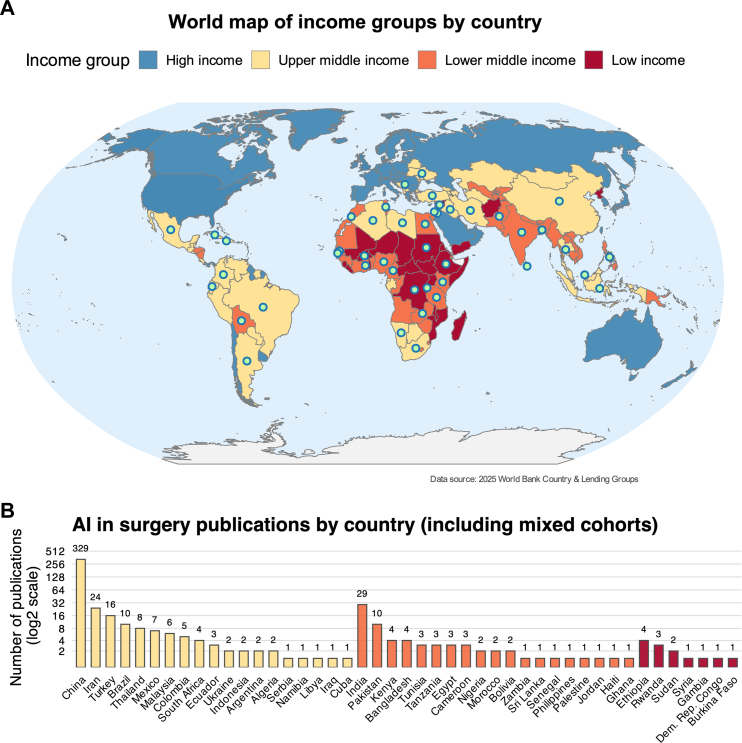
**Global distribution of Artificial Intelligence (AI) research in surgical care. (A)** World Bank income group classification of countries (2025). **(B)** Number of AI-related surgical publications by country, including studies with mixed cohorts, displayed on a log_2_ scale.

**Fig. 3:**
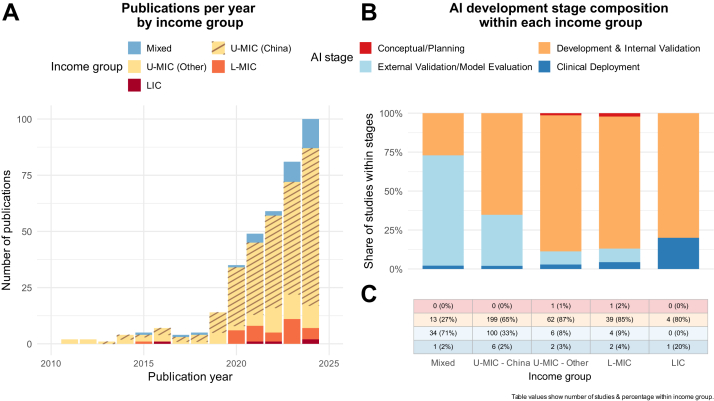
**Trends and maturity of Artificial Intelligence (AI) research in surgical care across income groups. (A)** Annual number of publications from 2010 to 2025, stratified by World Bank income group. **(B & C)** Proportional distribution of AI development stages within each income group, classified according to the Organisation for Economic Co-operation and Development (OECD) framework.

Most studies (n = 376, 79·1%) originated from upper-middle-income countries (U-MICs), with the overwhelming majority from China (n = 305, 64·2%). A further 71 studies (14·9%) originated from the remaining 17 other U-MIC countries. Only 46 studies (9·7%) were conducted in lower-middle-income countries (L-MICs) and just 5 (1·1%) in low-income countries (LICs). The remaining 48 studies (10·1%) included mixed populations spanning across multiple income settings. The global distribution of included studies is shown in Fig. 2. The annual number of publications has increased steadily since 2018, though this growth has been driven largely by studies from China (Fig. 3A).

Retrospective observational designs predominated, accounting for 325 studies (68·4%). Prospective observational studies were less frequent (n = 40, 8·4%), while randomised controlled trials were rare (n = 9, 1·9%). Across all settings, single-centre studies represented 277 records (58·3%) compared to 198 multi-centre studies (41·7%). Single-centre designs were particularly common in L-MICs (38/46, 82·6%) and LICs (3/5, 60·0%).

### Surgical domains and surgical care stages

General surgery was the most frequently represented specialty (n = 162, 34·1%), with most studies conducted in U-MIC-China (n = 116) and fewer in other U-MICs (n = 18), L-MICs (n = 9), mixed settings (n = 19), and none in LICs. Cardiothoracic surgery accounted for 56 studies (11·8%), followed by ophthalmological surgery with 39 studies (8·2%). Orthopaedic (n = 25, 5·3%), neurosurgery (n = 20, 4·2%), and spinal surgery (n = 20, 4·2%) were also represented. Obstetric or gynaecological surgery (n = 15, 3·2%) and paediatric surgery (n = 18, 3·8%) were notably less represented. A full breakdown of all specialties, including smaller fields such as urology, vascular, and ENT, is provided in Table 1.

**Table 1:** Characteristics of included studies on artificial intelligence in surgical care across income groups.

Study characteristics	Mixed cohort	U-MIC (China)	U-MIC (Other)	L-MIC	LIC	Total
Total number of studies N (%)	48 (10·1)	305 (64·2)	71 (14·9)	46 (9·7)	5 (1·1)	475
Surgical specialty						
General surgery	19 (39·6)	116 (38·0)	18 (25·4)	9 (19·6)	–	162 (34·1)
Obstetric or Gynaecological surgery	2 (4·2)	7 (2·3)	1 (1·4)	4 (8·7)	1 (20·0)	15 (3·2)
Urology	4 (8·3)	3 (1·0)	–	–	–	7 (1·5)
Transplantation surgery	1 (2·1)	10 (3·3)	5 (7·0)	3 (6·5)	–	19 (4·0)
Cardiothoracic surgery	3 (6·2)	39 (12·8)	10 (14·1)	3 (6·5)	1 (20·0)	56 (11·8)
Vascular surgery	–	5 (1·6)	2 (2·8)	1 (2·2)	–	8 (1·7)
Neurosurgery	1 (2·1)	12 (3·9)	4 (5·6)	3 (6·5)	–	20 (4·2)
Spinal surgery	–	17 (5·6)	1 (1·4)	2 (4·3)	–	20 (4·2)
Orthopaedic surgery	1 (2·1)	21 (6·9)	2 (2·8)	1 (2·2)	–	25 (5·3)
Plastic and Reconstructive surgery	1 (2·1)	3 (1·0)	3 (4·2)	2 (4·3)	–	9 (1·9)
Oral and Maxillofacial surgery	2 (4·2)	5 (1·6)	1 (1·4)	5 (10·9)	–	13 (2·7)
ENT surgery	–	14 (4·6)	1 (1·4)	1 (2·2)	–	16 (3·4)
Ophthalmological surgery	7 (14·6)	19 (6·2)	5 (7·0)	7 (15·2)	1 (20·0)	39 (8·2)
Paediatric surgery	3 (6·2)	7 (2·3)	5 (7·0)	3 (6·5)	–	18 (3·8)
Cross specialty	4 (8·3)	27 (8·9)	13 (18·3)	2 (4·3)	2 (40·0)	48 (10·1)
Study design						
Observational (Retrospective)	30 (62·5)	224 (73·4)	51 (71·8)	18 (39·1)	2 (40·0)	325 (68·4)
Observational (Prospective)	4 (8·3)	28 (9·2)	5 (7·0)	3 (6·5)	–	40 (8·4)
Randomised control trial	–	6 (2·0)	–	2 (4·3)	1 (20·0)	9 (1·9)
Other (Focus on model development)	14 (29·2)	47 (15·4)	15 (21·1)	23 (50·0)	2 (40·0)	101 (21·3)
Study centres						
Single centre	4 (8·3)	175 (57·4)	57 (80·3)	38 (82·6)	3 (60·0)	277 (58·3)
Multiple centres	44 (91·7)	130 (42·6)	14 (19·7)	8 (17·4)	2 (40·0)	198 (41·7)
Stage of surgical care						
Pre-operative	18 (37·5)	133 (43·6)	24 (33·8)	24 (52·2)	1 (20·0)	200 (42·1)
Intra-operative/Intra-procedural	6 (12·5)	27 (8·9)	4 (5·6)	2 (4·3)	–	39 (8·2)
Post-operative	20 (41·7)	134 (43·9)	36 (50·7)	19 (41·3)	3 (60·0)	212 (44·6)
Training/Simulation	3 (6·2)	4 (1·3)	3 (4·2)	1 (2·2)	–	11 (2·3)
Service/workflow monitoring	1 (2·1)	7 (2·3)	4 (5·6)	–	1 (20·0)	13 (2·7)
AI domain						
Clinical data-based predictive modelling	28 (58·3)	175 (57·4)	49 (69·0)	24 (52·2)	2 (40·0)	278 (58·5)
Image-based AI	12 (25·0)	66 (21·6)	15 (21·1)	12 (26·1)	3 (60·0)	108 (22·7)
Signal-based AI	1 (2·1)	3 (1·0)	1 (1·4)	3 (6·5)	–	8 (1·7)
Text/NLP-based AI	1 (2·1)	12 (3·9)	3 (4·2)	3 (6·5)	–	19 (4·0)
Genomics/molecular data AI	1 (2·1)	4 (1·3)	–	–	–	5 (1·1)
Multimodal AI	4 (8·3)	41 (13·4)	1 (1·4)	4 (8·7)	–	50 (10·5)
Service/workflow AI	1 (2·1)	4 (1·3)	2 (2·8)	–	–	7 (1·5)
AI development stage						
Conceptual/Planning	–	–	1 (1·4)	1 (2·2)	–	2 (0·4)
Development & internal validation	13 (27·1)	199 (65·2)	62 (87·3)	39 (84·8)	4 (80·0)	317 (66·7)
External validation	34 (70·8)	100 (32·8)	6 (8·5)	4 (8·7)	–	144 (30·3)
Clinical deployment	1 (2·1)	6 (2·0)	2 (2·8)	2 (4·3)	1 (20·0)	12 (2·5)

**Abbreviations**: **AI**, Artificial intelligence; **ENT**, Ear, nose, and throat; **LIC**, Low-income country; **L-MIC**, Lower-middle-income country; **NLP**, Natural language processing; **RCT**, Randomised controlled trial; **U-MIC**, Upper-middle-income country.

In terms of surgical care stage, most studies focused on post-operative care (n = 212, 44·6%) and pre-operative care (n = 200, 42·1%). Intra-operative studies, including examples of intra-procedural AI such as endoscopic procedures were less frequent (n = 39, 8·2%). Only 13 studies (2·7%) addressed service or workflow monitoring, and 11 (2·3%) involved training or simulation. Pre- and post-operative studies were widely distributed across all income groups, while intra-operative and monitoring studies were rarely reported in L-MICs or LICs.

### AI categories and development stages

Clinical data–based predictive modelling was the most common AI implementation approach, reported in 278 studies (58·5%). Image-based AI was also widely used (n = 108, 22·7%), with the majority originating from China (n = 66). Multimodal AI was identified in 50 studies (10·5%), again predominantly from higher-income settings. Other categories, including text/NLP-based AI (n = 19, 4·0%), appeared only infrequently (Table 1).

Most studies reported findings from development and internal validation (n = 317, 66·7%). External validation was performed in 144 studies (30·3%), but this was again largely concentrated in the mixed cohort studies (n = 34/144, 23·8%) and China (n = 100/144, 69·4%). Clinical deployment was reported in only 12 studies (2·5%). External validation, external model evaluation, and deployment were almost entirely absent in L-MICs and LICs, where research activity was overwhelmingly limited to the development and internal validation stage, Fig. 3.

### Studies reporting clinical deployment

Across the review, clinical deployment was rare (n = 12; 2·5%).^18^, ^19^, ^20^, ^21^, ^22^, ^23^, ^24^, ^25^, ^26^, ^27^, ^28^, ^29^ Clinical deployment was defined as use of an AI system within a real patient care pathway, with outputs available at the point of care and capable of informing a clinical action. In most cases, deployment reflected pilot or trial-period implementation embedded within routine services and evaluated prospectively, rather than fully integrated, sustained use at scale.

The deployed studies clustered into three practical patterns: (i) real-time procedural support during endoscopy (recognition systems used during colonoscopy or gastroscopy), (ii) screening and triage deployments in which AI outputs directly triggered referral pathways (most commonly diabetic retinopathy programmes), and (iii) patient-facing peri-operative tools supporting education or post-operative management (e.g. nutrition support and thyroid follow-up). The characteristics of these studies are summarised in Table 2.

**Table 2:** Characteristics of studies reporting clinical deployment of AI in surgical care across LMIC groups.

Study information	Study design sample size	Surgical specialty	Stage of surgical care + AI domain	Notes on deployment
Mixed cohort				
Schnabel et al. 2023^18^ India & Switzerland	Multi centre observational n = 19	Paediatric Surgery	Pre-operative Image-based AI	AI-driven workflow generating 3D-printable cleft lip palate plates used for patient treatment
U-MIC—China				
Lin et al. 2019^19^	Multi centre RCT n = 350	Ophthalmological Surgery	Pre-operative Image-based AI	AI platform provided diagnosis + treatment recommendations used in care
Wang et al. 2020^20^	Single centre RCT n = 1046	General Surgery	Intra-operative Image-based AI	Real-time computer-aided detection to increase adenoma detection during colonoscopy
Zhang et al. 2023^21^	Single centre observational n = 221	General Surgery	Post-operative Text/NLP-based AI	Automated medication dosage adjustment post-thyroid surgery + patient guidance
An et al. 2024^22^	Multi centre RCT n = 32	General Surgery	Training/Simulation Image-based AI	Real-time AI for upper GI endoscopy training, supporting blind-spot monitoring + lesion diagnosis
Yao et al. 2024^23^	Multi centre RCT n = 685	General Surgery	Training/Simulation Image-based AI	AI assistance during novice performed colonoscopy, reducing adenoma miss rate
Zhang et al. 2025^24^	Single centre RCT n = 75	Urology	Pre-operative Text/NLP-based AI	AI-based education programme on urolithiasis reduced preoperative anxiety
U-MIC–Other				
Ruamviboonsuk et al. 2022^25^ Thailand	Multi centre observational n = 7651	Ophthalmological Surgery	Pre-operative Image-based AI	AI enabled screening for diabetic retinopathy and macular oedema + referral recommendations embedded in screening workflow
Malherbe et al. 2025^26^ South Africa	Single centre observational n = 1617	General Surgery	Pre-operative Image-based AI	Breast AI integrated with clinical breast examination to guide targeted care decisions
L-MIC				
Abramoff et al. 2023^27^ Bangladesh	Single centre RCT n = 494	Ophthalmological Surgery	Pre-operative Image-based AI	Autonomous AI exam used during visits, changing clinic flow/time
Zahid et al. 2023^28^ Pakistan	Singel centre RCT n = 61	Paediatric Surgery	Post-operative Text/NLP-based AI	AI-based mobile app used to support dietary planning/tracking after paediatric surgery
LIC				
Mathenge et al. 2022^29^ Rwanda	Multi centre RCT n = 136	Ophthalmological Surgery	Pre-operative Image-based AI	AI-supported screening with immediate referral feedback to patients (vs delayed), affecting referral uptake

**Abbreviations**: **AI**, Artificial intelligence; **LIC**, Low-income country; **L-MIC**, Lower-middle-income country; **LMICs**, Low- and middle-income countries; **NLP**, Natural language processing; **RCT**, Randomised controlled trial; **U-MIC**, Upper-middle-income country.

Collectively, the maturity of deployment should be interpreted cautiously. The evidence is best interpreted as early-stage implementation, with limited reporting on sustainability, scale-up, and post-study maintenance. Where authors discussed scale-up explicitly, constraints included infrastructure readiness, training burden, and cost-effectiveness.^26^

## Discussion

This scoping review identified 475 primary studies evaluating the development, validation, and deployment of AI in surgical care across LMICs. Geographic distribution was uneven, with nearly three-quarters of studies originating from middle income countries in Asia, particularly China, India and Iran, while low income regions such as sub-Saharan Africa and parts of Latin America were notably underrepresented. Most studies were retrospective and focused on model development with internal validation, highlighting that the current evidence base remains at an early or exploratory stage.

Although previous studies have addressed the topic of AI in global surgery, this is the first large scale review with an emphasis on mapping the maturity of the AI evidence from LMIC settings. While research activity in HICs has expanded rapidly and has begun to progress toward external validation and clinical deployment, this review demonstrates that work in LMICs remains concentrated in the early phases of development. Prior work has mostly consisted of editorials,^11^^,^^30^ policy proposals,^31^ or reviews limited to specific subspecialties.^32^, ^33^, ^34^, ^35^ Through a broad and protocol driven search, this scoping review has demonstrated the geographical differences in AI adoption, identified a lack of research beyond internal validation, and highlighted important barriers and facilitators to the implementation of surgical AI in LMICs.

An important barrier to AI implementation is that these solutions do not inherently generalise across different populations. Ideally, models developed in HICs would work just as well in low-resource settings, sparing LMICs the burden of building systems from scratch. However, when put into practice such models commonly underperform in new environments even after adaptation.^36^, ^37^, ^38^ Considerable variation in language, culture, ethnicity, and disease burden can also exist within LMICs. This further complicates model transferability within these regions. The above underscore the necessity of diverse, representative datasets and rigorous local validation to ensure AI tools are reliable and effective in varied LMIC settings.

Building on the need for diverse and representative datasets, a critical challenge for LMICs is the lack of infrastructure to reliably capture and manage high-quality clinical data.^39^^,^^40^ Limited adoption of electronic health records hampers access to structured clinical information at scale, particularly in surgical settings where perioperative data is often recorded in unstructured ways. Additionally, LMICs conduct far fewer surgeries (approximately 877 per 100,000 population compared to 10,000 in high-income countries) reducing the volume of operative data available for model development.^41^^,^^42^ Without significant investment in digital infrastructure to support routine data collection and storage, the potential benefits of AI in LMICs risk remaining out of reach.

Developing accurate, context-specific AI models is only half the challenge; deploying them within LMIC health systems comes with its own complexities. The review identified very few studies that progressed to deployment, underscoring the difficulty of real-world clinical adoption. LMIC health facilities can be situated in areas lacking reliable access to the internet and compatible digital devices, which can hinder deployment at the point of care.^10^^,^^43^ In addition, premature digital health strategies can also hinder integration, raising concerns that AI solutions may be underused or abandoned after pilot phases. Tools introduced without reliable infrastructure and stakeholder buy-in risk being perceived as burdensome rather than helpful by clinical staff on the ground.

Workforce limitations, including shortages of healthcare workers and low levels of digital and AI literacy, further constrain implementation efforts.^44^^,^^45^ Additional challenges arise from concerns about legal responsibility, data privacy, and potential loss of clinical autonomy, as evidenced by surveys of LMIC clinicians and patients.^46^, ^47^, ^48^, ^49^, ^50^ Finally, it is often unclear who will fund and maintain these tools after initial development. Even when tools are free to the end user and operate on a non-profit basis, they still incur ongoing expenses for maintenance, hosting, and support.^9^^,^^43^ Put simply, even the most accurate models will have no impact if the health systems lack the capacity to adopt and integrate them.

Despite the barriers, the literature also highlights several facilitators to the implementation of AI in LMIC settings. Mobile-phone based AI tools provide a valuable opportunity for overcoming traditional infrastructure barriers in resource-limited settings. By leveraging the widespread ownership and use of smartphone devices among healthcare workers, mobile device solutions can enable essential functions such as image capture, remote consulting, and asynchronous education without relying on hospital servers or advanced computer systems. Where electronic health systems are lacking, mobile images captured by phones are already being used by clinicians as a tool to document and review surgical cases.^28^^,^^40^^,^^51^, ^52^, ^53^, ^54^, ^55^ AI-powered video analysis of simple mobile footage has also been used to provide actionable feedback to surgical trainees, a major benefit in areas with limited teaching faculty.^56^ The findings reflect how innovation in LMICs often comes from adaptation of widely available tools rather than large institutional systems.

In addition, the review reveals a wealth of emerging LMIC-led AI research that addresses the unique challenges faced in LMIC surgical care. For example, predictive models are being developed to assist frontline teams in identifying high-risk patients, especially where follow-up systems are weak or fragmented.^57^, ^58^, ^59^ These applications offer opportunities for early use cases. Participatory design, involving co-creation with local stakeholders, further enhances the relevance and acceptance of these AI solutions, supporting their long-term viability. Together, these factors represent key facilitators that support equitable and effective AI adoption and implementation in LMICs. An overall summary of the barriers and facilitators discussed is presented in Table 3.

**Table 3:** Barriers and facilitators to the implementation of AI in surgical care in LMIC health systems, grouped by thematic category.

Category	Barriers	Facilitators
Model and data challenges	- AI models developed in HICs often fail to generalise to LMICs due to population health differences, clinical workflows, and data availability. - Even within LMICs, variations in language, culture, and disease patterns reduce model transferability. - Limited local data availability and quality due to low EHR adoption, small sample sizes, incomplete records, and lower surgical volumes in LMICs constrain training and validation.	- Growing LMIC-led AI research is expanding access to diverse, representative datasets that reflect local epidemiological and clinical contexts. - Demonstrated emphasis on local data collection, model fine-tuning, and validation enhances context-specific accuracy and relevance.
Infrastructure and technology	- Many LMIC health facilities lack reliable internet, stable power, and compatible devices, limiting point-of-care AI deployment. - Underdeveloped digital health strategies hinder integration and lead to underuse or abandonment of AI tools after pilot phases. - Tools introduced without adequate infrastructure or stakeholder buy-in may be seen as burdensome rather than supportive.	- Mobile-based AI tools leverage widespread healthcare worker mobile phone ownership to circumvent infrastructure barriers. - Mobile applications facilitate clinical documentation and feedback by utilizing readily available devices within healthcare settings. - Locally developed predictive models are being piloted to identify high-risk postoperative patients, enhancing care continuity in settings with limited follow-up infrastructure.
Workforce	- Healthcare worker shortages limit the capacity to implement AI; without sufficient staff, AI tools cannot be applied. - Low digital and AI literacy among clinicians makes AI deployment more challenging. - Concerns about legal responsibility, data privacy, and clinical autonomy reduce trust and slow adoption.	- The literature documents targeted training initiatives that enhance digital and AI literacy among healthcare professionals. - Participatory co-design methodologies actively involve clinicians, fostering tool alignment with local workflows and strengthening user trust.
Governance, trust, and sustainability	- Lack of clear governance and legal frameworks complicates accountability and data privacy concerns. - Uncertainty over who will fund ongoing maintenance, hosting, and support threatens long-term sustainability. - Even free tools incur operational costs, and without designated responsibility, tools risk abandonment.	- Collaborative and open-source AI development initiatives demonstrate improved transparency, reproducibility, and multi-stakeholder engagement. - Evidence highlights that open-source platforms facilitate cooperative innovation and accelerate equitable AI integration.

Interpretation of the findings should take into account the constraints of both the underlying evidence and the review. The scarcity of data from certain LMIC regions, especially sub-Saharan Africa and Latin America, restricts the generalizability of results across the diverse spectrum of LMICs. Finally, the heterogeneity of AI technologies and surgical contexts also limited comparative analysis, reflecting the rationale for an exploratory scoping review. Taken together, the findings highlight the need for more focused future studies that consider setting-specific requirements and the relative maturity of the AI the systems under investigation.

Looking to the future, realizing the potential advantages of AI in LMIC surgical settings will require foundational investments and evaluation in the settings the tools are intended to be used. Strengthening local data infrastructure is critical for accurate AI development and evaluation. Collaborative efforts like the Global Burden of Disease study have illustrated how coordinated data capture can yield insights into LMIC health challenges.^60^ Along infrastructure enhancement, validation across diverse LMIC surgical settings will be essential to establish their utility and effectiveness. To validate AI tools effectively, it is important to test them within existing clinical care pathways.

Strengthening the broader AI ecosystem in LMICs is equally vital and requires co-developing policy frameworks, governance standards, and sustainable funding models with local stakeholder involvement to ensure relevance and ownership. The WHO Digital Health Guidelines offer the foundations for ethical and governance standards adaptable to diverse LMIC contexts.^61^ Concurrently, embedding responsible AI principles like fairness, transparency, accountability, and community engagement is critical to address ethical challenges and foster trust. Involving local communities in AI design and oversight will ensure alignment with social and cultural values, promoting equitable and ethical deployment.^62^

A strength of this review is its breadth, providing a high-level map of AI research across surgical care in LMIC settings. The review also builds on prior work by characterising the literature using an OECD-aligned AI maturity framework. This provides a clearer picture of the progress in developing, validating, and deploying AI in surgical care across LMICs. Further strengths include granular characterisation by surgical subspecialty and stage of surgical care, and the inclusion of a dedicated analysis of the cohort of studies reporting clinical deployment. Lastly, we incorporated a qualitative synthesis of barriers and facilitators, shaped by authors with lived clinical experience of delivering surgical care in LMIC environments, to ensure that the implementation narrative remained grounded and relevant.

The breadth of this review is also its main limitation, as it restricts the depth of the review. It was not feasible to conduct full-text qualitative synthesis across all included studies. For the discussion around facilitators and barriers, our review focused on studies from L-MIC and LIC settings to prioritise the most resource-constrained contexts, recognising that barriers and enabling conditions in U-MIC settings may differ and that potentially transferable insights from these settings may sit outside the LIC/LMIC-focused synthesis. To keep the review achievable and aligned to our surgical decision-making focus, we also excluded studies using radiology-only or histology-only inputs, even where outputs might plausibly influence care, which may have led to under-capture of some relevant work. Restricting inclusion to full-text reports available in English was a further limitation. While our searches did not exclude studies by language, and only 8 (1·4%) of full texts were excluded for this reason, this exclusion may still under-represent LMIC work published in local-language journals or in sources not indexed by major databases.

Heterogeneity within countries and within income group classifications is another inherent limitation. Although we have taken steps to improve interpretability by disaggregating U-MIC studies into China and other U-MIC settings, national income grouping may still mask meaningful sub-national variation in infrastructure and resources, therefore limiting generalisability. Finally, studies classified as “clinical deployment” were mostly pilot or trial-period implementations, and there was limited reporting on sustainability, scale-up, and post-study maintenance.

This scoping review maps the emerging but uneven landscape of AI implementation in surgical care across LMICs, with research from LICs markedly underrepresented. The recent rising volume of research suggests growing interest and activity, but significant challenges remain including limited data infrastructure and barriers to model generalisability. Addressing these issues will require coordinated efforts to build robust local data capture systems, embed rigorous validation across diverse populations, and strengthen research networks grounded in responsible AI principles. Focusing on these priorities, future research can support the responsible adoption of AI to enhance surgical care across the world.

## Contributors

GK and ADV conceived and designed the study (conceptualisation, methodology). ADV, GK, and SJT conducted the literature search, screening, and data charting (data curation, investigation). All authors–ADV, SJT, SK, TSRK, BK, SRK, CW, GK—contributed to the analysis and interpretation of the data (formal analysis). ADV and GK drafted the initial manuscript (writing—original draft). All authors–ADV, SJT, SK, TSRK, BK, SRK, CW, GK–contributed to critical revision of the manuscript for important intellectual content (writing—review & editing). GK and CW provided overall supervision (supervision, project administration). GK and ADV had full access to all the data and verified the underlying data. All authors–ADV, SJT, SK, TSRK, BK, SRK, CW, GK–read and approved the final version of the manuscript. GK and CW contributed equally as senior authors.

## Data sharing statement

The extracted summary data supporting the findings of this review can be obtained from the corresponding author upon reasonable request and without restriction. All data will be made available from the date of publication.

## Editor note

The Lancet Group takes a neutral position with respect to territorial claims in published maps and institutional affiliations.

## Declaration of interests

All authors declare no financial or personal relationships that could have influenced the work reported in this paper.
