# Supplementary material for: Artificial intelligence in surgical care within low-income and middle-income countries: a scoping review of development, validation, and deployment
Source: eClinicalMedicine. 2026 Mar 16;94:103836. doi: 10.1016/j.eclinm.2026.103836 (PMC13011077; doi:10.1016/j.eclinm.2026.103836)
Supplement: Appendix [file mmc1.docx]

Supplementary Material

Artificial intelligence in surgical care within low- and middle-income countries: a scoping review of development, validation, and deployment

Aashobanaa Duraisaminathan Valli, MRes^1^, Samuel James Tingle, MRCS^1, 2, 3^, Sofia Kazerouni, MBBS^1, 2, 3^, Tanissha Sanjay Raj Kalpana, MBBS^4^, Bishow Karki, MRCS^5, 6^, Stephen R Knight, PhD^7,8^, Prof Colin Wilson, PhD^1, 2, 3†^, Georgios Kourounis, MRCS^1, 2, 3 †^

**Appendix 1.** Search strategies

**Appendix 2.** Large language model assisted data extraction methodology

**Supplementary Table S1**. PICOS criteria for study inclusion and exclusion

## **Appendix 1.** Search strategies

### Pubmed search

( ( "surgeon*"[Title/Abstract] OR “surger*"[Title/Abstract] OR “surgical*"[Title/Abstract] OR "operative"[Title/Abstract] OR "preoper*"[Title/Abstract] OR “pre-oper*"[Title/Abstract] OR “pre oper*"[Title/Abstract] OR "intraoper*"[Title/Abstract] OR “intra-oper*"[Title/Abstract] OR “intra oper*"[Title/Abstract] OR “postoper*"[Title/Abstract] OR “post-oper*"[Title/Abstract] OR “post oper*"[Title/Abstract] OR "perioper*"[Title/Abstract] OR “peri-oper*"[Title/Abstract] OR “peri oper*"[Title/Abstract] OR "surgical procedure*"[Title/Abstract] OR “surgical intervention*"[Title/Abstract] OR “surgical care"[Title/Abstract] )

AND

( "artificial intelligence"[Title/Abstract] OR “neural network"[Title/Abstract] OR "deep learning"[Title/Abstract] OR “computer vision"[Title/Abstract] OR “machine learning"[Title/Abstract] OR "natural language processing"[Title/Abstract])

AND

( ( "low income"[Title/Abstract] OR “middle income"[Title/Abstract] OR “low-income"[Title/Abstract] OR “middle-income"[Title/Abstract] OR “LMIC"[Title/Abstract] OR “developing countr*"[Title/Abstract] OR “resource-limited"[Title/Abstract] OR “low-resource"[Title/Abstract] OR “low resource"[Title/Abstract] OR “resource-constrained"[Title/Abstract] OR “developing world"[Title/Abstract] OR “global surgery"[Title/Abstract] OR"Africa South of the Sahara"[MeSH] OR “Asia, Southeastern"[MeSH] OR “Latin America"[MeSH])

OR

("Afghanistan"[Title/Abstract] OR “North Korea"[Title/Abstract] OR “Somalia"[Title/Abstract] OR “Burkina Faso"[Title/Abstract] OR “Liberia"[Title/Abstract] OR “South Sudan"[Title/Abstract] OR “Burundi"[Title/Abstract] OR “Madagascar"[Title/Abstract] OR “Sudan"[Title/Abstract] OR “Central African Republic"[Title/Abstract] OR “Malawi"[Title/Abstract] OR “Syrian Arab Republic"[Title/Abstract] OR “Chad"[Title/Abstract] OR “Mali"[Title/Abstract] OR “Togo"[Title/Abstract] OR "Democratic Republic of Congo"[Title/Abstract] OR "Mozambique"[Title/Abstract] OR "Uganda"[Title/Abstract] OR "Eritrea"[Title/Abstract] OR "Niger"[Title/Abstract] OR "Yemen"[Title/Abstract] OR "Gambia"[Title/Abstract] OR "Rwanda"[Title/Abstract] OR "Guinea-Bissau"[Title/Abstract] OR "Sierra Leone"[Title/Abstract] )

OR

("Angola"[Title/Abstract] OR "India"[Title/Abstract] OR "Papua New Guinea"[Title/Abstract] OR "Bangladesh"[Title/Abstract] OR "Jordan"[Title/Abstract] OR "Philippines"[Title/Abstract] OR "Benin"[Title/Abstract] OR "Kenya"[Title/Abstract] OR "São Tomé and Principe"[Title/Abstract] OR "Bhutan"[Title/Abstract] OR "Kiribati"[Title/Abstract] OR "Senegal"[Title/Abstract] OR "Bolivia"[Title/Abstract] OR "Kyrgyz Republic"[Title/Abstract] OR "Solomon Islands"[Title/Abstract] OR "Cambodia"[Title/Abstract] OR "Lao PDR"[Title/Abstract] OR "Sri Lanka"[Title/Abstract] OR "Cameroon"[Title/Abstract] OR "Lebanon"[Title/Abstract] OR "Tajikistan"[Title/Abstract] OR "Comoros"[Title/Abstract] OR "Lesotho"[Title/Abstract] OR "Tanzania"[Title/Abstract] OR "Republic of Congo"[Title/Abstract] OR "Mauritania"[Title/Abstract] OR "Timor-Leste"[Title/Abstract] OR "Côte d'Ivoire"[Title/Abstract] OR "Micronesia"[Title/Abstract] OR "Tunisia"[Title/Abstract] OR "Djibouti"[Title/Abstract] OR "Morocco"[Title/Abstract] OR "Uzbekistan"[Title/Abstract] OR "Egypt"[Title/Abstract] OR "Myanmar"[Title/Abstract] OR "Vanuatu"[Title/Abstract] OR "Eswatini"[Title/Abstract] OR "Namibia"[Title/Abstract] OR "Viet Nam"[Title/Abstract] OR "Vietnam"[Title/Abstract] OR "Ghana"[Title/Abstract] OR "Nepal"[Title/Abstract] OR "West Bank"[Title/Abstract] OR "Gaza"[Title/Abstract] OR "Palestine"[Title/Abstract] OR "Guinea"[Title/Abstract] OR "Nicaragua"[Title/Abstract] OR "Zambia"[Title/Abstract] OR "Haiti"[Title/Abstract] OR "Nigeria"[Title/Abstract] OR "Zimbabwe"[Title/Abstract] OR "Honduras"[Title/Abstract] OR "Pakistan"[Title/Abstract])

OR

("Albania"[Title/Abstract] OR "Equatorial Guinea"[Title/Abstract] OR "Moldova"[Title/Abstract] OR "Algeria"[Title/Abstract] OR "Fiji"[Title/Abstract] OR "Mongolia"[Title/Abstract] OR "Argentina"[Title/Abstract] OR "Gabon"[Title/Abstract] OR "Montenegro"[Title/Abstract] OR "Armenia"[Title/Abstract] OR "Georgia"[Title/Abstract] OR "North Macedonia"[Title/Abstract] OR "Azerbaijan"[Title/Abstract] OR "Grenada"[Title/Abstract] OR "Paraguay"[Title/Abstract] OR "Belarus"[Title/Abstract] OR "Guatemala"[Title/Abstract] OR "Peru"[Title/Abstract] OR "Belize"[Title/Abstract] OR "Indonesia"[Title/Abstract] OR "Samoa"[Title/Abstract] OR "Bosnia and Herzegovina"[Title/Abstract] OR "Iran"[Title/Abstract] OR "Serbia"[Title/Abstract] OR "Botswana"[Title/Abstract] OR "Iraq"[Title/Abstract] OR "South Africa"[Title/Abstract] OR "Brazil"[Title/Abstract] OR "Jamaica"[Title/Abstract] OR "St. Lucia"[Title/Abstract] OR "Cabo Verde"[Title/Abstract] OR "Kazakhstan"[Title/Abstract] OR "St. Vincent and the Grenadines"[Title/Abstract] OR "China"[Title/Abstract] OR "Kosovo"[Title/Abstract] OR "Suriname"[Title/Abstract] OR "Colombia"[Title/Abstract] OR "Libya"[Title/Abstract] OR "Thailand"[Title/Abstract] OR "Cuba"[Title/Abstract] OR "Malaysia"[Title/Abstract] OR "Tonga"[Title/Abstract] OR "Dominica"[Title/Abstract] OR "Maldives"[Title/Abstract] OR "Türkiye"[Title/Abstract] OR "Turkey"[Title/Abstract] OR "Dominican Republic"[Title/Abstract] OR "Marshall Islands"[Title/Abstract] OR "Turkmenistan"[Title/Abstract] OR "Ecuador"[Title/Abstract] OR "Mauritius"[Title/Abstract] OR "Tuvalu"[Title/Abstract] OR "El Salvador"[Title/Abstract] OR "Mexico"[Title/Abstract] OR "Ukraine"[Title/Abstract]

)))NOT Animals[MeSH]

### Scopus search

TITLE-ABS-KEY( ("surgeon*" OR "surger*" OR "surgical*" OR "preoper*" OR "pre-oper*" OR "pre oper*" OR "intraoper*" OR "intra-oper*" OR "intra oper*" OR "postoper*" OR "post-oper*" OR "post oper*" OR "perioper*" OR "peri-oper*" OR "peri oper*" OR "surgical procedure*" OR "surgical intervention*" OR "surgical care")

AND

("artificial intelligence" OR "neural network" OR "deep learning" OR "computer vision" OR "machine learning" OR "natural language processing")

AND

("low income" OR "middle income" OR "low-income" OR "middle-income" OR "LMIC" OR "developing countr*" OR "resource-limited" OR "low-resource" OR "low resource" OR "resource-constrained" OR "developing world" OR "global surgery" OR "Africa South of the Sahara" OR "Asia, Southeastern" OR "Latin America" OR "Afghanistan" OR "North Korea" OR "Somalia" OR "Burkina Faso" OR "Liberia" OR "South Sudan" OR "Burundi" OR "Madagascar" OR "Sudan" OR "Central African Republic" OR "Malawi" OR "Syrian Arab Republic" OR "Chad" OR "Mali" OR "Togo" OR "Democratic Republic of Congo" OR "Mozambique" OR "Uganda" OR "Eritrea" OR "Niger" OR "Yemen" OR "Gambia" OR "Rwanda" OR "Guinea-Bissau" OR "Sierra Leone" OR "Angola" OR "India" OR "Papua New Guinea" OR "Bangladesh" OR "Jordan" OR "Philippines" OR "Benin" OR "Kenya" OR "São Tomé and Principe" OR "Bhutan" OR "Kiribati" OR "Senegal" OR "Bolivia" OR "Kyrgyz Republic" OR "Solomon Islands" OR "Cambodia" OR "Lao PDR" OR "Sri Lanka" OR "Cameroon" OR "Lebanon" OR "Tajikistan" OR "Comoros" OR "Lesotho" OR "Tanzania" OR "Republic of Congo" OR "Mauritania" OR "Timor-Leste" OR "Côte d'Ivoire" OR "Micronesia" OR "Tunisia" OR "Djibouti" OR "Morocco" OR "Uzbekistan" OR "Egypt" OR "Myanmar" OR "Vanuatu" OR "Eswatini" OR "Namibia" OR "Viet Nam" OR "Vietnam" OR "Ghana" OR "Nepal" OR "West Bank" OR "Gaza" OR "Palestine" OR "Guinea" OR "Nicaragua" OR "Zambia" OR "Haiti" OR "Nigeria" OR "Zimbabwe" OR "Honduras" OR "Pakistan" OR "Albania" OR "Equatorial Guinea" OR "Moldova" OR "Algeria" OR "Fiji" OR "Mongolia" OR "Argentina" OR "Gabon" OR "Montenegro" OR "Armenia" OR "Georgia" OR "North Macedonia" OR "Azerbaijan" OR "Grenada" OR "Paraguay" OR "Belarus" OR "Guatemala" OR "Peru" OR "Belize" OR "Indonesia" OR "Samoa" OR "Bosnia and Herzegovina" OR "Iran" OR "Serbia" OR "Botswana" OR "Iraq" OR "South Africa" OR "Brazil" OR "Jamaica" OR "St. Lucia" OR "Cabo Verde" OR "Kazakhstan" OR "St. Vincent and the Grenadines" OR "China" OR "Kosovo" OR "Suriname" OR "Colombia" OR "Libya" OR "Thailand" OR "Cuba" OR "Malaysia" OR "Tonga" OR "Dominica" OR "Maldives" OR "Türkiye" OR "Turkey" OR "Dominican Republic" OR "Marshall Islands" OR "Turkmenistan" OR "Ecuador" OR "Mauritius" OR "Tuvalu" OR "El Salvador" OR "Mexico" OR "Ukraine"))

AND

NOT TITLE-ABS-KEY("rat" OR "mouse" OR "mice" OR "murine" OR "animal study" OR "rabbit" OR "pig")

### Web of science search

TS=( ("surgeon*" OR "surger*" OR "surgical*" OR "preoper*" OR "pre-oper*" OR "pre oper*" OR "intraoper*" OR "intra-oper*" OR "intra oper*" OR "postoper*" OR "post-oper*" OR "post oper*" OR "perioper*" OR "peri-oper*" OR "peri oper*" OR "surgical procedure*" OR "surgical intervention*" OR "surgical care")

AND

("artificial intelligence" OR "neural network" OR "deep learning" OR "computer vision" OR "machine learning" OR "natural language processing")

AND

("low income" OR "middle income" OR "low-income" OR "middle-income" OR "LMIC" OR "developing countr*" OR "resource-limited" OR "low-resource" OR "low resource" OR "resource-constrained" OR "developing world" OR "global surgery" OR "Africa South of the Sahara" OR "Asia, Southeastern" OR "Latin America" OR "Afghanistan" OR "North Korea" OR "Somalia" OR "Burkina Faso" OR "Liberia" OR "South Sudan" OR "Burundi" OR "Madagascar" OR "Sudan" OR "Central African Republic" OR "Malawi" OR "Syrian Arab Republic" OR "Chad" OR "Mali" OR "Togo" OR "Democratic Republic of Congo" OR "Mozambique" OR "Uganda" OR "Eritrea" OR "Niger" OR "Yemen" OR "Gambia" OR "Rwanda" OR "Guinea-Bissau" OR "Sierra Leone" OR "Angola" OR "India" OR "Papua New Guinea" OR "Bangladesh" OR "Jordan" OR "Philippines" OR "Benin" OR "Kenya" OR "São Tomé and Principe" OR "Bhutan" OR "Kiribati" OR "Senegal" OR "Bolivia" OR "Kyrgyz Republic" OR "Solomon Islands" OR "Cambodia" OR "Lao PDR" OR "Sri Lanka" OR "Cameroon" OR "Lebanon" OR "Tajikistan" OR "Comoros" OR "Lesotho" OR "Tanzania" OR "Republic of Congo" OR "Mauritania" OR "Timor-Leste" OR "Côte d'Ivoire" OR "Micronesia" OR "Tunisia" OR "Djibouti" OR "Morocco" OR "Uzbekistan" OR "Egypt" OR "Myanmar" OR "Vanuatu" OR "Eswatini" OR "Namibia" OR "Viet Nam" OR "Vietnam" OR "Ghana" OR "Nepal" OR "West Bank" OR "Gaza" OR "Palestine" OR "Guinea" OR "Nicaragua" OR "Zambia" OR "Haiti" OR "Nigeria" OR "Zimbabwe" OR "Honduras" OR "Pakistan" OR "Albania" OR "Equatorial Guinea" OR "Moldova" OR "Algeria" OR "Fiji" OR "Mongolia" OR Argentina" OR "Gabon" OR "Montenegro" OR "Armenia" OR "Georgia" OR "North Macedonia" OR "Azerbaijan" OR "Grenada" OR "Paraguay" OR "Belarus" OR "Guatemala" OR "Peru" OR "Belize" OR "Indonesia" OR "Samoa" OR "Bosnia and Herzegovina" OR "Iran" OR "Serbia" OR "Botswana" OR "Iraq" OR "South Africa" OR "Brazil" OR "Jamaica" OR "St. Lucia" OR "Cabo Verde" OR "Kazakhstan" OR "St. Vincent and the Grenadines" OR "China" OR "Kosovo" OR "Suriname" OR "Colombia" OR "Libya" OR "Thailand" OR "Cuba" OR "Malaysia" OR “Tonga" OR "Dominica" OR "Maldives" OR "Türkiye" OR "Turkey" OR "Dominican Republic" OR "Marshall Islands" OR "Turkmenistan" OR "Ecuador" OR "Mauritius" OR "Tuvalu" OR "El Salvador" OR "Mexico" OR "Ukraine") )

NOT TS=("rat" OR "mouse" OR "mice" OR "murine" OR "animal study" OR "rabbit" OR "pig")

AND

DT=(Article OR Review OR "Practice Guidelines")

## **Appendix 2.** Large language model assisted data extraction methodology

### Summary of the approach

Given the large number of eligible studies, we adopted the help of a large language model (LLM) to perform the first pass of data extraction using the information in the study titles and abstracts. The aim of the approach was to facilitate timely extraction of predefined study characteristics while maintaining human oversight and accountability. A predefined structured prompt was developed and iteratively refined, and the final prompt used for data extraction is provided in full at the end of this appendix.

### Technical requirements for the approach

All eligible study records were exported into a comma-separated values (CSV) file, with each row representing a single study and containing the study title and abstract text. The CSV file was accessed using Visual Studio Code (VS Code) with GitHub Copilot enabled, which provided access to the LLM (Claude Sonnet 4.0). The LLM was applied directly to the CSV content using the structured prompt described below.

### Development steps before finalising prompt and methodology

An iterative development process was used to refine both the extraction workflow and the final prompt structure. The principle was to rely on information contained within the title and abstract to populate predefined extraction fields using a structured prompt. During piloting, we also explored extraction from full-text PDFs. This substantially increased processing time and computational cost (GitHub Copilot credits). We therefore restricted automated extraction to titles and abstracts, with full texts reserved for human extraction.

To minimise hallucinations by the LLM, the prompt explicitly included a “Not enough information” response option for all relevant fields, which was used when the title and abstract did not provide sufficient detail. These cases highlighted where subsequent human extraction was needed.

During testing, we observed that performance was improved when a human investigator manually extracted data for an initial small number of records (five studies) before initiating automated extraction. We also found that processing records in batches of approximately 20 rows at a time produced more reliable outputs; larger batch sizes were associated with system instability or obvious extraction errors.

### Evaluating the approach

Following automated extraction, a human reviewer assessed the accuracy of all extracted data fields by comparing the LLM outputs against the full-text articles. Where discrepancies were identified between the automated extraction and human assessment, the study was independently reviewed by a second author and discrepancies were resolved by consensus. The frequency of required human corrections for each data extraction field is summarised below.

| **Data extraction field** | **Studies requiring human input (n)** | **Percentage (%)** |
| --- | --- | --- |
| **Country** | 117 | 22.5% |
| **Surgical specialty** | 60 | 11.5% |
| **Study design** | 80 | 15.4% |
| **Study centres** | 27 | 5.2% |
| **Stage of surgical care** | 45 | 8.7% |
| **AI domain** | 41 | 7.9% |
| **AI development stage** | 43 | 8.3% |
| **Sample size** | 126 | 24.2% |
| **Any field requiring human input (per study)** | 327 | 62.9% |
| **Any field requiring human input (per extraction field)** | 539 | 13.0% |

### Data extraction prompt

For each row that has an individual study, read the title and abstract information so that you can extract the following information.

If the title and abstract do not provide enough information respond with "Not enough information", I don't want you hallucinating results.

**Country**

The country (or countries, if multiple) of the patient population for the study. Do not rely on author locations alone to define this information.

Sometimes the author locations are not the same as the patient locations (ie HIC researchers in the US or Canada, developing a tool with cohorts of patients in a LMIC like Rwanda - if this is the case I want the country to be labelled as Rwanda)

**StudyDesign - One of the following**

Descriptive (survey/qualitative)

Observational (not specified) - this is for timing not specified

Observational (retrospective)

Observational (prospective) - use this for both exclusively prospective studies as well as mixed studies that have both retrospective and prospective cohorts

Randomised control trial

Other ("specifyhere") - replace "specifyhere" with a description of the other type of study

Insufficient information - this is the option if you cannot make an assessment

**SurgicalSpecialty - One of the following**

General Surgery
 Endocrine Surgery
 Breast Surgery
 Transplantation Surgery
 Vascular Surgery
 Urology
 Cardiothoracic Surgery
 Orthopaedic Surgery
 Neurosurgery
 Spinal Surgery

Obstetric or Gynaecological Surgery

Plastic and Reconstructive Surgery

Pediatric Surgery

ENT Surgery

Ophthalmological Surgery

Oral and Maxillofacial Surgery

Cross Specialty

**CategoryOfAI - One of the following**

Clinical data-based predictive modeling (traditional tabular data)

Image-based AI (radiological, photographic, endoscopic, histological)

Signal-based AI (physiological signals, wearables, biosensors)

Text/NLP-based AI (clinical notes, reports)

Genomics/molecular data AI (transcriptomics, proteomics, bioinformatics)

Multimodal AI (combining 2+ data types)

Operational/workflow AI (scheduling, resource allocation)

Training/simulation AI

**SampleSize**

Size of the sample of whatever they used (ie patients, videos, photographs, surgeons, etc)

Here are some examples as samples of what I am expecting in this column

514 laparoscopic images

892 patients

1,107 videos from 87 surgeons

**Centres - One of the following**

Single centre

Multiple centres

**RCT - one of the following depending on if the study is a randomised control trail or not**

Yes

No

**SurgicalCareStage**

Pre-operative

Intra-operative

Post-operative

Training/simulation

Operational monitoring

**AI_lifecycle_stage - one of the Values (in order of increasing maturity, mirroring the OECD** **FRAMEWORK FOR THE CLASSIFICATION OF AI SYSTEMS)**

Conceptual/Planning: When the study is primarily about planning, comparing existing models, or theoretical work without developing new models

Development & Internal Validation: When a model is developed and validated using techniques like cross-validation, train/test splits, or internal validation within the same dataset

External Validation: When a model is developed in one dataset/institution and then tested on a completely different external dataset/institution

Clinical Deployment: When an AI system is actually deployed and used in clinical practice to make real decisions

Operational Monitoring - AI system deployed with ongoing performance monitoring

External Model Use or Evaluation - Study uses or evaluates existing AI systems (not developed by authors)

Use the information that you extract to write the correct information into the csv file please.

Write all info into the csv using quotations so that the commas in the answers don't create new columns by mistake. that will break the file.

## **Supplementary Table S1**. PICOS criteria for study inclusion and exclusion

|  | **Inclusion Criteria** | **Exclusion Criteria** |
| --- | --- | --- |
| **Population** | - Surgical patients or surgical care  - Settings in low- and middle-income countries (LMICs) (World Bank classification)  - Accept studies with a mix of patients in LMIC and HIC | - Studies with populations exclusively from HIC |
| **Intervention** | - Use of AI technologies in any aspect of surgical care:  - Preoperative planning  - Intraoperative support  - Postoperative care  - Complication management  - Surgical decision support  - Surgical education  - Robotic surgery where adjunct AI tools are incorporated  - Multimodal and or perioperative AI models that incorporate radiological or histological information alongside non-imaging data | - Robotic surgery without AI components  - Simulation or virtual reality tools without AI integration  - Prediction models that do not involve machine learning or AI beyond classical statistical modelling (regression)  - AI models that use only radiological or histological information |
| **Comparator** | - Not required (all study designs considered) | - Not applicable |
| **Outcomes** | - Description or evaluation of AI implementation, applications, outcomes, feasibility, or barriers in surgical settings | - Studies without outcome data or implementation descriptions (e.g., editorials without data) |
| **Study Design** | - Primary research studies  - Guidelines or policy analyses providing direct evidence of AI applications in surgery in LMIC settings | - Protocols  - Meeting abstracts  - Commentaries, editorials, or opinion pieces without primary data or analysis  - Bibliometric studies  - Review articles  - Retracted articles  - Preprints |
